# Safety and therapeutic efficacy of the switch to maraviroc+darunavir/ritonavir in HIV/HCV coinfected patients: initial results from GUSTA study

**DOI:** 10.7448/IAS.17.4.19818

**Published:** 2014-11-02

**Authors:** Roberta Gagliardini, Barbara Rossetti, Claudia Bianco, Stefano Rusconi, Manuela Colafigli, Roberta Prinapori, Daniela Francisci, Alessandra Fantauzzi, Giancarlo Orofino, Francesca Vignale, Simona Di Giambenedetto, Andrea De Luca

**Affiliations:** 1 Clinical Infectious Diseases, Catholic University of Sacred Heart, Rome, Italy; 2 Infectious Diseases Unit, Azienda Ospedaliera Universitaria Senese, Siena, Italy; 3 Infectious Disease Unit, DIBIC Luigi Sacco, University of Milan, Milano, Italy; 4 Infectious Dermatology and Allergologic Unit, IFO S Gallicano, Roma, Italy; 5 Clinica Malattie Infettive, Università Degli Studi di Genova, Genova, Italy; 6 Dipartimento di Medicina, Università di Perugia, Perugia, Italy; 7 Dipartimento Medicina Clinica, Sapienza Università di Roma, Roma, Italy; 8 Diseases Unit A, Amedeo di Savoia Hospital, Torino, Italy; 9 Clinic of Infectious Diseases, University G. D'Annunzio, Chieti, Italy

## Abstract

**Introduction:**

HIV/HCV coinfection is a risk factor for hepatic injury in patients receiving HAART and previous studies support a favourable effect of antiretroviral regimens including maraviroc (MVC) on the course of coinfection compared with other antiretroviral drugs. There are few observations about MVC use in simplified treatment of coinfected patients.**Objective**: To evaluate the efficacy and the safety of simplification to darunavir (DRV)/ritonavir (r)/maraviroc (MVC) in virologically HIV-suppressed patients and to explore the effect of simplified treatment on coinfected patients.

**Material and Methods:**

GUSTA study is a randomized two arms trial that compares the switch to DRV/r/MVC with standard HAART with three drugs. The study enrols patients with HIV-1 RNA<50cp/mL>6 months, R5 tropism, CD4>200 cells/mm. Survival analysis was used to analyze factors associated to time-to a single viral load (VL) over 50cp/mL and FIB-4>1.45.

**Results:**

We included 62 patients with at least the 24 week follow-up for FIB-4 analysis: males 75.8%, heterosexuals 48.4%, HCV+12.9% median age 48.3 years (IQR41.1;53.5), time from HIV diagnosis 11.0 years (IQR7.3;16.7), CD4 cells 659/mm (IQR478;882), nadir CD4 203/mm (IQR115;286), FPR 46 (IQR30;70), baseline (BL) FIB-4 1.11 (IQR0.75;1.35). At BL no differences were observed in the two arms, except for platelets (−34.96 109/L, in the study arm, p=0.028) and CD4 at nadir (−70cell/µL, p0.051). After 24 weeks a significant reduction in total bilirubin (TB) (−0.55 mg/dL, p=0.025) and alkaline phosphatase(AP) (−12.96 UI/L, p=0.002) was observed in the study group. A statistically significant difference in mean change of TB (0.61 mg/dL, p=0.016) and AP (13.23 UI/L, p=0.04) at 24 week between control and study group was observed. No grade 3/4 transaminase elevation was observed for any patient even if HIV/HCV coinfected and receiving MVC. A single HCV negative patient in the control arm had grade 3 bilirubin increase. Including all patients with at least one follow-up HCV status was not associated with an increased risk of detectable VL (n=114, 4072 person-week-follow-up [IQR12;51.6]), nor with FIB-4>1.45 (n=98, 3513 person-week-follow-up [IQR11.4;50.9]).

**Conclusions:**

The initial results from GUSTA study show that ART-regimen including MVC did not increase the incidence of adverse events or severe laboratory liver abnormalities in HIV-1-infected patients with or without HCV coinfection. Coinfected patients did not show an increased risk of failure on simplification treatment with MVC/DRV/r.

**Table 1:** Factors associated with FIB-4>1.45 at Cox regression analysis

	Univariate analysis			Multivariate analysis		
	
Factors associated with FIB-4>1.45 at Cox regression analysis	HR	CI	p	HR	CI	p
Boosted protease inhibitors previous use	2.554	0.853; 7.648	0.094	1.315	0.413; 4.185	0.643
Raltegravir previous	3.896	1.133; 13.405	0.031	6.122	1.278; 29.328	0.023
BL FIB-4	74.08	7.91; 694.18	<0.001	129.232	9.792; 1705.564	<0.001
Hcv positive	1.011	0.275; 3.713	0.986	0.485	0.112; 2.103	0.334
Study group	2.086	0.839; 5.185	0.113	0.958	0.354; 2.589	0.932

